# Entropy Method of Road Safety Management: Case Study of the Russian Federation

**DOI:** 10.3390/e24020177

**Published:** 2022-01-25

**Authors:** Artur I. Petrov

**Affiliations:** Department of Road Transport Operation, The Institute of Transport, Industrial University of Tyumen, 625027 Tyumen, Russia; ArtIgPetrov@yandex.ru; Tel.: +7-(912)-079-19-91

**Keywords:** entropy, quantitative assessment, Shannon’s relative entropy, system orderliness, road safety management, Russian Federation

## Abstract

Within the framework of this paper, the author’s entropy method of road safety management in large-sized systems is considered. The road safety management system in the Russian Federation, the largest country in the world, was selected for this case study. The purpose of the article is to present the opportunities and methodology of the use of quantitative assessments of the orderliness of the road accident rate formation process in regional transport systems for road safety management. Orderliness, in other words, systemic anti-chaos, can be quantified using the C. Shannon informational entropy *H*. The article consists of the results of the issue’s state analysis; methodology of assessment of the orderliness of the road accident rate formation process based on the using of the cause-and-effect chain; entropic method of the road safety management in large-scale systems, in particular, the algorithm of management of regional road safety in Russia taking into account the level of its entropic orderliness; and examples of the quantitative evaluation of the orderliness of regional road safety provision systems in Russia. The key results of the research are spatio-temporal patterns of the change of the orderliness of the road safety provision systems in the Russian Federation in 2004–2020. Based on the results, conclusions and recommendations about the practical application of the entropic method of road safety management in large federal states with complex administrative structures were formulated. These results give an idea of the possibilities of the usage of entropic approaches in road safety management to assess the orderliness of the regional transport systems and the advantages of the entropic method over other managerial methods.

## 1. Introduction

Socio-technical systems are characterized by the highest level of complexity. A huge number of system elements and a great variety of connections between them determine a high level of the probability of chaos in such systems. That is why many regulatory documents and technical and technological procedures are being developed to manage their functioning. Their primary goal is to regulate and control the implementation of individual actions by the process participants in order to reduce the number of possible process outcomes. In addition, it is important to control the quality implementation of the requirements of these regulations. The ultimate goal of management is to transfer the controlled system to the desired, targeted, more or less unambiguous, planned state, which means reducing the level of chaos in the system.

The main purpose of road traffic safety systems is to reduce the likelihood of incidents (in the form of road traffic accidents) in the functioning of the transport system. Analytical work with statistical data characterizing the road accident rate does not allow us to fully understand the mechanisms of its formation. Only the use of a deeper entropy analysis can help in substantive assessment of what is happening on a large scale, for example, regional road safety systems.

Over the last 125 years of active motorization, in the field of road safety, five paradigms have successively replaced one another–approaches, practices, and knowledge systems generally recognized by the entire scientific community [1,2]. However, the process of rebuilding road safety systems is extremely heterogeneous in terms of space and time. Even in the regions of one country, very different road safety practices related to different paradigms simultaneously coexist. This largely depends on the characteristics of the socio-economic development of the regions [3]. To a greater extent, this is typical for large-size countries. Russia, with its 17.5 million km^2^ of land and with more than 190 peoples and nationalities inhabiting it, is a vivid example of a state with a wide variety of public administration practices.

According to N.V. Zubarevich [4]: “today there exist four different versions of Russia at the same time–metropolitan Russia, Russia of large cities, Russia of small and medium-sized cities, and rural Russia”, each of which is fundamentally different not only in the socio-economic standard of living but also in people’s way of life, mentality, and habits. At the everyday level, this is manifested in a variety of behavioral practices, including on the road when driving a vehicle [5,6].

At the state level, the issues of ensuring road traffic safety in Russia have received great attention since 2006. It was then that the first (2006–2012) and the second (2013–2020) [7] federal target programs on road safety were adopted and began to be implemented. Starting from 2018, road safety management processes in Russia are based on the targets of the Road Safety Strategy in the Russian Federation for 2018–2024 [8].

According to this document, by 2024, the level of Human Risk (*HR*) (one of the main status indicators of road safety) in Russia should be reduced to 4 road deaths/100 thousand people. In 2020, Human Risk in Russia was *HR* = 11.01 road deaths/100 thousand people [9], which means that within 4 years (2021–2024), it would be necessary to reduce the road death rate as the final outcome of road traffic accidents in Russia by 2.75 times or by 175%.

Recall that Human Risk (*HR*) is calculated according to (1) [1]:

(1) $$HR=\frac{N_{RA\;deaths}}{\left(P/100,000\right)},$$

where

*N_RA deaths_*—the number of deaths in road accidents, people;
*P*—population, people;
100,000—conversion factor.

Analysis shows that this is hardly possible [10,11].

Examples of other world-leading countries in the field of road safety indicate that it took them 10–36 years to solve this problem (Table 1) [12,13]. Initially, Russia planned to solve this problem within 5 years (2020–2024). However, it quickly became clear that the long journey of other countries to *HR* = 4 road deaths/100 thousand people is quite justified.

In 2020, the country’s leadership realized that the task could not be solved by 2024. In this regard, by the Decree of the President of the Russian Federation No. 474 of 21 July 2020, the deadline for achieving the goal in *HR* = 4 road deaths/100 thousand people was shifted from 2024 to 2030 [14].

Note that having already reached the level of *HR* = 4 road deaths/100 thousand people, Germany, Spain, and the Netherlands have remained at this level for almost a decade and have not yet been able to improve the situation in the field of road safety. Maybe this is due to the internal policies of these countries, which are quite liberal and do not overly restrict the freedoms of citizens. Perhaps, without a total infringement on civil liberties, the task of a significant increase in the road safety level cannot be solved. In any case, this is evidenced by the analysis of the data in Table 1. International practices do not have examples of such rates of Human Risk reduction that were set for Russia by the Road Safety Strategy in the Russian Federation (2018–2024).

Does Russia have a chance to greatly accelerate the course of history and take advantage of the already existing developments of foreign authors in order to realize extremely ambitious goals? By implementing what solutions can this be achieved? What is the specificity of road safety formation in different regions of the Russian Federation? Can entropy analysis help in understanding the processes of road accident rate formation? An attempt to answer these questions was made by the author within the framework of this paper.

## 2. Analysis of the Issue–Theoretical Foundations

### 2.1. Generally Accepted Road Safety Management Methods

**Figure 1:**
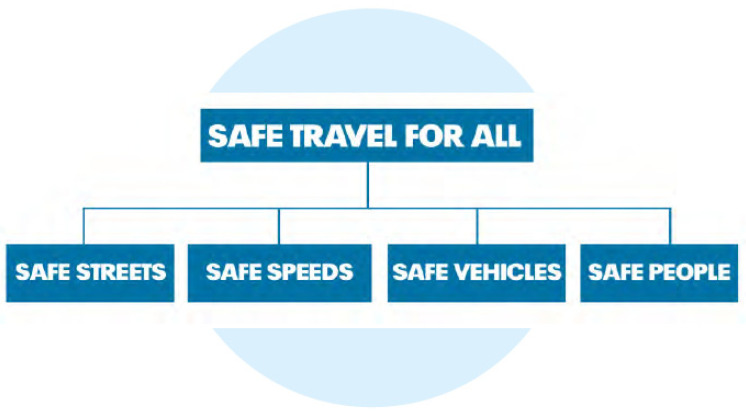
Four approaches to achieving the goal of zero road deaths (Vision Zero) [15].

The global goal of road safety management is to minimize the number of fatalities in road accidents down to zero. In fact, we must talk about management as a search for ways to reduce the likelihood of the formation of emergency situations in the transport system, i.e., about reducing the level of systemic chaos through the implementation of managerial decisions aimed at improving the orderliness of the transport system or at least the part of it that specializes in road safety. The Swedish program Vision Zero [15], adopted by the Swedish Parliament in October 1997, declares the following main approaches to achieving this goal (Figure 1): improving the road transport infrastructure; controlling the speed limit; improving vehicle safety; working with the mentality of people; and the formation of safe stereotypes of behavior among road users.

The main methods of road safety management correspond to the above basic approaches. It is often organizationally difficult to implement all approaches in practice at the same time due to a shortage of resources of all types. Therefore, most often, we can see the choice of only one approach as a priority [16]. For example, in Russia, in recent years (2019–2021), an approach to improve the road transport infrastructure is the method of choice [17]. At the physical level, this is expressed not only in improving the quality of highways [18,19] but also in complicating traffic flow regulation [20,21].

We know [22,23,24] that a much more effective method for increasing road safety is changing the stereotypes of typical behavior of road users. The analysis [25,26] shows that statistically significant effects of changes in people’s behavior are formed under conditions of creating an atmosphere of reward for the required behavioral imperative and, conversely, punishment in case of violations of the prescribed rules. It has been statistically proven that traffic rules are observed to the fullest extent possible in countries with the highest rates of fines for their violation [27]. However, not only fines affect the behavior of road users but also the peculiarities of the national mentality [28], which has been developing over the centuries [29]. An analysis of the research results [30,31] showed that the degree of influence of such an approach to improving road safety as control over the speed limit with subsequent punishment for violations varies in different countries [30] and even in the regions of one country [31].

### 2.2. Heterogeneity of the Conditions for the Functioning of Russian Regional Transport Systems as a Challenge to the Quality Road Safety Management on the Part of Federal Bodies

**Figure 2:**
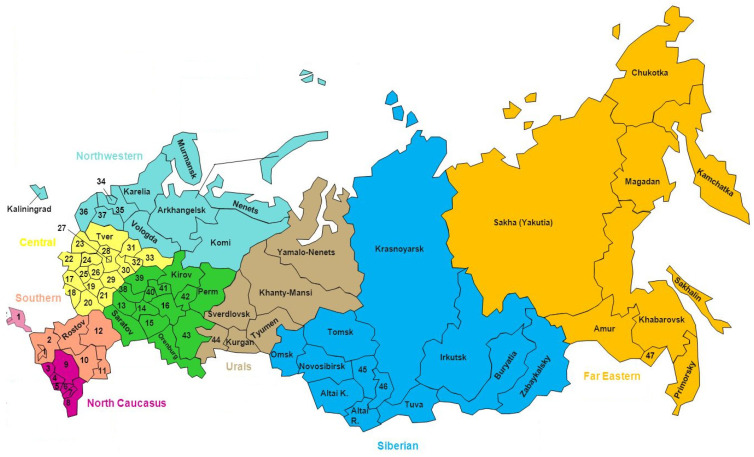
Administrative division of the Russian Federation into 8 Federal Districts, (highlighted on the map in different colors) and 85 subjects of the Federation (regions).

Russia is a federal state governed from a single center on the basis of a unified legislation. The Russian Federation unites 85 subjects (82 territories and 3 cities of federal subordination) (Figure 2). The constituent entities of the Russian Federation are united in eight Federal Districts: Central, Northwestern, Southern, North Caucasian, Volga, Ural, Siberian, and Far Eastern. The best climate, maximum population density, and, accordingly, better living conditions are typical for the Central and Southern Federal Districts. The Ural Federal Districts, Siberian Federal Districts, and Far Eastern Federal Districts encompass territories with a harsh climate; low population density and low degree of socio-economic development of the territories are quite consistent with very harsh living conditions.

The transport system of a country is a set of regional transport systems which in turn unite regional subsystems of the road transport infrastructure, vehicle fleet, regulation of the transport process, ensuring road safety, and many other subsystems that support the transport process. The development of regional transport systems in Russia is very heterogeneous–from a level comparable to EU countries (for example, the Moscow region) to a level close to that of African countries (for example, the Republic of Tyva).

In the Russian Federation, as of 31 December 2020, there were 58,993 thousand vehicles in the road network with a total length of 1553.6 thousand km. During operation in 2020, 145,073 road accidents occurred in the Russian Federation [32] (in Russia, the concept of road traffic accidents applies to cases with the dead and injured; other incidents on the road–with only material damage–are not included in the official statistics).

**Figure 3:**
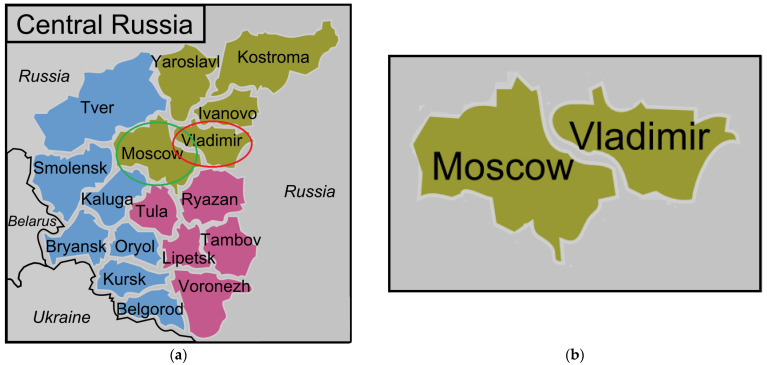
Geographical location of the compared Russian regions. (**a**) Central Federal District of the Russian Federation; (**b**) Geographically adjacent Moscow and Vladimir regions.

Within the territory of the country, road accidents are distributed extremely heterogeneously, which largely depends on the density of the population, its socio-economic situation, the degree of development of the road transport infrastructure, the quality of the vehicle fleet, and many other factors. This heterogeneity can be easily identified even in case of neighboring regions, for example, the Moscow and Vladimir regions (Figure 3).

In the adjacent Moscow and Vladimir regions, both the living conditions of the population [33] and the characteristics of road accident rates [9,32] are qualitatively different (Table 2).

There are many similar examples for the Russian Federation–the Republic of Tatarstan and the Republic of Mordovia, Tyumen and Kurgan regions, etc. Among the 85 constituent entities of the Russian Federation, there are virtually no identical regions comparable in terms of the level of socio-economic development.

As a result, different budgetary provision and different levels of quality of life of the population have formed in the regions of the country. This diversity is also manifested in the field of road safety (Table 2).

Perhaps, it can be argued that the heterogeneity of the conditions for the functioning of regional transport systems is a serious challenge to the quality road safety management on the part of federal bodies.

At the same time, it becomes clear that federal bodies have few tools for assessing the qualitative state of the actual level of development of regional road safety systems. This toolkit for road safety management (in the form of an approach or a method) is extremely important precisely for large and complex countries such as Russia.

### 2.3. The Problem of the Correct Choice of Indicators of Quality Road Safety Management

The problem of effective road safety management within a large country such as the Russian Federation is the absence of a clear methodology and a set of correct indicators for assessing the quality of such management. Today, the goal of road safety management is clear–to achieve the planned level of *HR* = 4 road deaths/100 thousand people by 2030 in the entire country. However, it is unclear how to organize the processes of achieving this goal for numerous regions in Russia (85 in total). The current approach is to use three absolute indicators to assess the road safety state in the regions of the country–the annual road accident number, the annual number of injured and dead in road accidents, and one relative indicator–the road accident severity, as well as a comparison of the current values of these characteristics with their values in previous time periods. This approach is not only ineffective but also methodologically incorrect. Analytical work on a comparative assessment of the road safety state in road transport systems varying in area, population, and the degree of development cannot and should not be carried out on the basis of the analysis of absolute indicators. This is incorrect [34], and experts are well aware of this. However, they have no other tools, although they are very interested in obtaining them.

### 2.4. Substantiation of the Choice for Characterizing the Quality of Road Safety Management by Information Entropy

In its modern interpretation, the high quality of process management or management of the functioning of complex systems is characterized by a low level of chaos of processes and a high probability of finding a system at a certain, given state [35]. The probability of finding a system at a given state is a function of information entropy [36].

The concept of entropy has a complex history. It was first introduced by R. Clausius in thermodynamics in 1865 to determine the measure of irreversible dissipation of energy, the measure of deviation of a real process from an ideal one [37]. Over the next one hundred and fifty years, the concept of entropy has become widely used in various scientific fields. As M.M. Kostic points out in [38]: «Entropy is the most used and often abused concept in science, but also in philosophy and society. Further confusions are produced by some attempts to generalize entropy with similar but not the same concepts in other disciplines … Von Neumann once remarked that “whoever uses the term «entropy» in a discussion always wins since no one knows what entropy really is, so in a debate one always has the advantage”. The historian of science and mathematician, Truesdell, explains in his essay of Method and Taste in Natural Philosophy: “Heads have split for a century trying to define entropy in terms of other things”».

**Figure 4:**
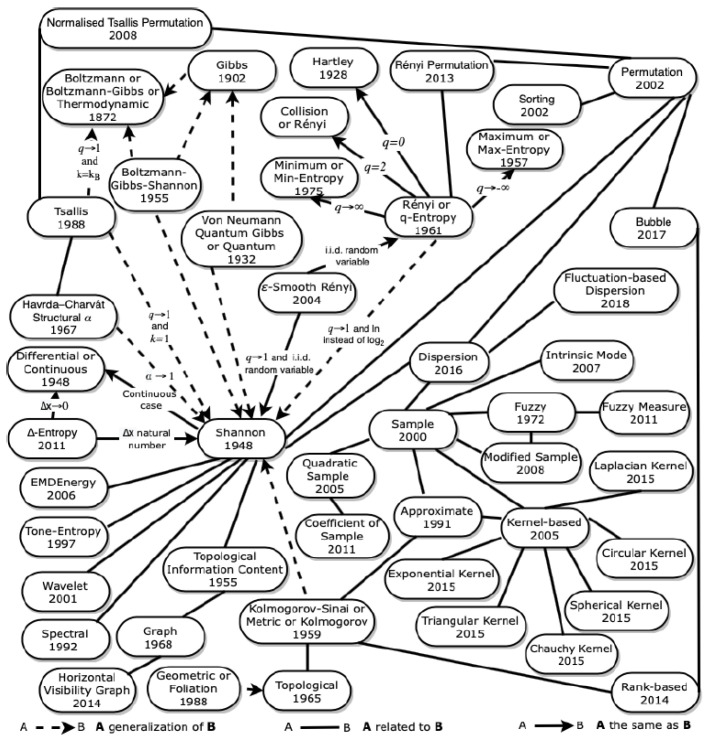
The Entropy Universe [39].

An in-depth analysis carried out by Portuguese authors [39] on entropy helped not only to formalize the history of the development of the concept of “entropy” but also to construct the so-called Entropy Universe (Figure 4).

The central place in it is occupied by Shannon entropy or information entropy [40]. It is information entropy that today dominates among other entropies. Statistical analysis by the team of authors M. Ribeiro et al. [39] showed that, in total, articles published in the Scopus peer-reviewed journals mention Shannon information entropy 34,751 times, which is about 49.2% of the total number of mentions of 40 other types of entropy (70,495 in total). Perhaps, the same conclusion can be made by visually assessing the central localization of Shannon entropy in the diagram in Figure 4 and the number of logical connections between Shannon entropy with other types of entropy.

Information entropy *H*, or Shannon entropy, characterizes the probability of a certain macroscopic state to be performed. The low level of entropy *H* means that there are far fewer ways to obtain a given macro-state from its microscopic ingredients. Such configurations are difficult to find, unusual, carefully organized, and rare.

### 2.5. Information Entropy of a System as a Basis for Analyzing the Quality of Road Safety Management: Orderliness as a Systemic Property

As applied to transport systems, information entropy *H* identifies the probability of a transport system being in a chaotic state, i.e., a condition for which the safety level is low. It is logical that under conditions of simultaneous interaction of a multitude of vehicles (their number depends on the scale of the system being evaluated), the probability of their safe interconnection in the process of movement is relatively small. However, this probability of a safe state of the transport system can be increased through the use of various instruments for regulating its functioning. Striking examples of such regulation are traffic rules, traffic light control systems for traffic flows, differentiation of traffic flows in space, and many other important methods of organizing traffic. In general, traffic management is a tool for reducing the information entropy of a transport system and bringing it into a state of order, i.e., increasing its orderliness. However, a person (driver or pedestrian) uses these tools and does not always follow the recommendations of restrictions and systems that regulate traffic processes. As a result, there is an increase in systemic chaos and disorganization of those processes that must be ordered.

The extent to which the problem of improving the system orderliness is solved in practice can ultimately be assessed by means of Shannon information entropy [40]. With regard to the quality of road safety management, Shannon entropy identifies the level of orderliness of the road safety system. The lower the value of information entropy *H*, the higher the orderliness of the system. The classical Shannon Formula (2) for the case of assessing the orderliness of the road safety system takes on the following meaning:

(2) $$H=-\sum_{i=1}^{n}w_{i}\cdot \ln w_{i}$$

where

*n*—the number of transfer links in the formation of a road accident rate (in our case *n* = 4) and $\omega_{i}$—the coefficients of significance or “weight” of each link in the formation of a road accident rate that meet the rate setting condition $\sum_{i=1}^{n}\omega_{i}=1$.

The number of links studied in terms of assessing the orderliness of the management process can be different and depend on the availability of statistics or on the formulation of the problem.

For the convenience of using this tool in order to conveniently compare the assessment of the quality of road safety management in different regions of the country, it is perhaps better to use the relative entropy indicator *H_n_* (3):

(3) $$H_{n}=H/H_{\max}=H/\ln(n)$$

The theoretical range of values of the relative entropy *H_n RSS_* of road safety systems *H_n RSS_* = [0; 1], however, in practice, *H_n RSS_* for the four-link process of road accident rate formation is most often in the range *H_n RSS_* = [0.65; 0.85] [41]. Lower values of the relative entropy *H_n RSS_* indicate a relatively high quality of road safety management and vice versa [41].

## 3. Methodology for Assessing the Orderliness of the Process of the Road Accident Rate Formation

Generally speaking, the target function of road safety management is to minimize all types of damage from road accidents, primarily direct damage associated with the death and injury of people in traffic accidents. In general, socio-economic damage in different countries of the world varies in the range from 0.8% to 4% of Gross Domestic Product (GDP) [42,43], and this value is determined by two main factors–the general level of road safety in the country and the level of the average cost of living of the citizens of this country [44]. These two aspects are interconnected by means of risk homeostasis, which is familiar to and accepted by the citizens of the country [45]. G.J.S. Wilde [45] believes that it is risk homeostasis that is the primary basis for understanding how the process of road accident rate is formed.

Without touching on the topic of assessing damage from road accidents in detail, we point out that many works are devoted to this topic [46,47,48]. Two aspects are important here: first, understanding that damage from road death is the highest in value and significantly exceeds material damage; and second, the ratio between the size of the vehicle fleet in the country, the number of road accidents, the number of victims in road accidents, and the proportion of fatalities among those injured in road accidents in different countries varies greatly. The same difference is typical for different regions of the same country and is determined by many circumstances of a socio-economic nature (Table 2).

Specifying the formulation of the target function of road safety management, we note the following: the general target function of road safety management at the federal level in large complex countries is to minimize the relative entropy *H_n RSS_* of the road safety formation process by maximizing the overall utility (positive Q) of the road accident rate formation process.

From a general philosophical point of view, it is impossible to look for a positive in road accidents, but let us consider the positive *Q* as a measure of the amount of information or a derivative of the entropy of the process under study.

**Figure 5:**
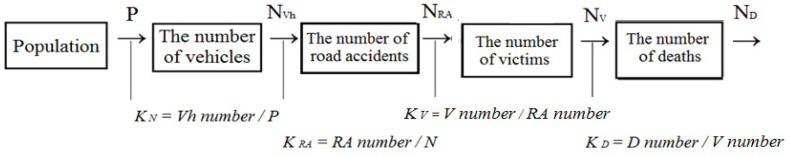
The cause–effect chain of road accident rate formation [49].

Formalization of the concept of “Road accident rate” in terms of the process description leads us to a cause–effect 4-link chain «*Population (P)—The number of vehicles (N_Vh_)—The number of road accidents (N_RA_)—The number of victims (N_V_)—The number of deaths (N_D_)*» [49] (Figure 5).

In this case (Figure 5), the process of road accident rate formation is considered as a set of four process links between the five main blocks of the process.

The road accident rate as a process can have many options. The distinction of the options is determined by the numerical values of the transition coefficients *K_i_* between the blocks of the cause–effect chain of the road accident formation process (*K_N_; K_RA_; K_V_; K_D_*). The positive of a separate link in the process *Qi* is associated with the transition coefficient *K_i_* by means of the relation (4):

(4) $$Q_{i}=\ln\left(1/K_{i}\right)\;\mathrm{if}\;{K_{i}}_{\;}<\;1\;\mathrm{or}\;Q_{i}=\ln\left(K_{i}\right)\;\mathrm{if}\;{K_{i}}_{\;}>\;1.\;$$

In general, the overall utility or positive of the process is identified as (5):

(5) $$Q=\ln\left(1/K_{HR}\right)=\ln\left(1/K_{N}\right)+\ln\left(1/K_{RA}\right)+\;\ln(K_{V})\;+\;\ln\left(1/K_{D}\right)=Q_{N}+Q_{RA}+\;Q_{V\;}+\;Q_{D},$$

where

*K_HR_*—the end-to-end path transmission factor *K_HR_ = D number/P*;
$Q_{N}=\ln\left(1/K_{N}\right)$—the share of the process positive attributed to the link “the number of vehicles”;
$Q_{RA}=\ln\left(1/K_{RA}\right)$—the share of the process positive attributed to the link “the number of road accidents”;
$Q_{D}=\ln\left(K_{v}\right)$—the share of the process positive attributed to the link “the number of victims”;
$Q_{D}=\ln\left(1/K_{D}\right)$—share of the process positive attributed to the link “the number of deaths”.

The use of the coefficient *K_HR_* in the model (4), i.e., the result of the ratio “The number of deaths *(N_D_*)/Population (*P*)” is predetermined by the physical meaning of this coefficient. *K_HR_* is a version of Human Risk (*HR*), proposed back in 1949 by R. Smeed [50,51], but in a slightly different dimension. Note that the higher the value of the positive, the higher the quality of the entire system of organization and management of road safety.

An important step in the methodology is to determine the structure of the weight coefficients $w_{i}$ for assessing the positive contribution *Q* of various links in the chain “Population (*P*)-< … >-The number of deaths (*N_D_*)”. The calculated weight values of the positive $w_{RA}$, $w_{V}$, and $w_{D}$ allows for solving the main problem of entropy analysis–assessing the degree of influence of various blocks (links) of the chain “Population (*P*)-< … >-The number of deaths (*N_D_*)” in the formation of the final accident rate.

The share of each link $w_{i}$ [52] of the cause–effect chain of road accident rate formation in the overall balance as (6):

(6) $$w_{i}=\frac{Q_{i}}{\sum_{i=1}^{4}Q_{i}}=\frac{Q_{i}}{\ln(1/K_{N})\;+\;\ln(1/K_{RA})\;+\;\ln(K_{V})\;+\;\ln(1/K_{D})}$$

The obtained estimates of the “weights” $w_{i}$ or the values of individual process links in the overall process are the starting material for the structural analysis of the process under study. Thus, relative entropy *H_n_* characterizes, first of all, the structure of the entire process of the road accident rate formation, rather than the ratio of the final result of the process–«The number of deaths (*N_D_*) by Population (*P*)». Quantitative estimates of the “weights” $w_{i}$ of the process links are basic data for determining the entropy characteristics according to Formulas (2) and (3).

The relative entropy *H_n RSS_* of the road accident rate formation process is a characteristic of the system orderliness and allows one to objectively judge the general situation in the field of road safety for the local system. Quantitative estimates of the relative entropy *H_n RSS_* of the road accident rate formation process can be used both for spatial analysis of the state of road safety systems and for assessing the dynamics of the process in time.

To specify the methodology of the assessment of the orderliness of the road accident rate formation process, we present it in the view of the formulated stages. It should be noted that three examples of such staged assessment are given in Section 5 of this paper.

The methodology consists of the sequential implementation of the next operations:

1. The determination of the values of transitional coefficients *K_i_* between the blocks of the cause-and-effect chain of the road accident rate formation process (*K_N_; K_RA_; K_V_; K_D_*).
2. The determination of the positive of the individual blocks of the road accident rate formation *Q_i_*. Partial positives *Q_i_* of individual blocks of the studied process are connected with the transitional coefficient *K_i_* of the cause-and-effect chain by relation (3).
3. The determination of the common positive *Q* by Formula (4).
4. The determination of the contribution $w_{i}$ of each block of the process *K_i_* into the common positive *Q* by Formula (5). The physical meaning of $w_{i}$ is the determination of «weight» or «significance» of an individual block of the cause-and-effect chain in the total result.
5. The determination of the values [$\ln\;(w_{i})$].
6. The determination of the values [$w_{i}$·$\ln\;(w_{i})$].
7. The calculation of the values of the C. Shannon informational entropy *H_RSS_* by the classic Formula (1).
8. The calculation of the values of the C. Shannon relative informational entropy *H_n RSS_* by Formula (2).

## 4. The Proposed Entropy Method for Road Safety Management in Large-Scale Systems

Road safety management in a large country such as the Russian Federation should be based on the use of a qualitative characteristic such as the relative entropy of the regional road safety system *H_n RSS_*.

**Figure 6:**
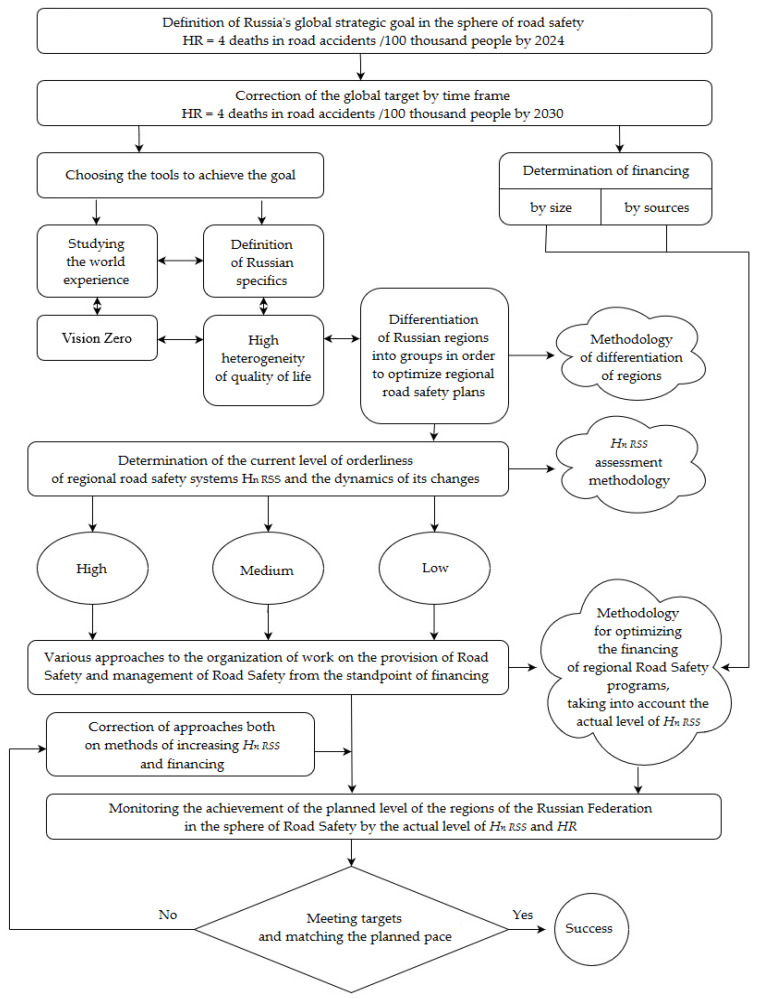
An algorithm for managing regional road safety in Russia, considering the level of its entropy orderliness.

The main purpose of the entropy method is the differentiation of regions of a large country into groups with high, average, and low levels of orderliness of road safety systems. Depending on this, the programs for financing the goals of improving road safety are configured (Figure 6).

The algorithm for managing regional road safety in Russia considering the level of its entropy orderliness implies the use of three individual methodologies–the methodology for differentiating regions by groups, the methodology for assessing the relative entropy *H_n RSS_* of the regional road safety system, and the methodology for optimizing the financing of regional programs for road safety considering the actual level of *H_n RSS_*.

The detailing of these three methods is a task for future research that is not included in the scope of this article. In this regard, we leave it to the next stage of the corresponding research.

Next, we consider specific examples of a quantitative assessment of the orderliness of road safety systems.

## 5. Examples of Quantitative Assessment of the Orderliness of the Federal Road Safety Management System in the Russian Federation and Some of Its Representative Regions

Table 3 shows an example of calculating (year 2020) the relative entropy *H_n RSS_* of the all-Russian road safety system. We used the 2020 Statistical Book (Informational and analytical review) of the Research Center of the State Traffic Safety Inspectorate of the Russian Federation [9] as the source of data.

**Figure 7:**
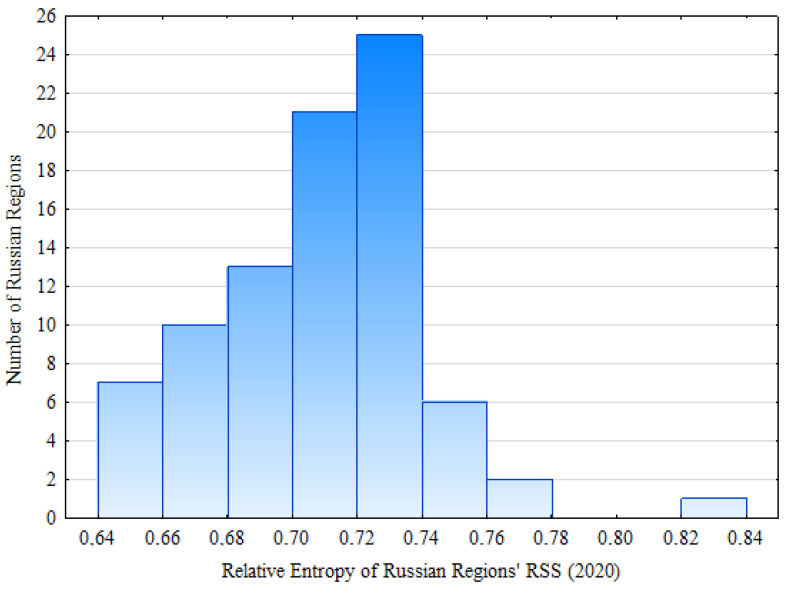
The distribution of the numerical values of the relative entropy *H_n RSS_* of regional road safety systems in 85 constituent entities of the Russian Federation (2020).

Figure 7 shows the distribution of the numerical values of the relative entropy *H_n RSS_* of regional road safety systems in 85 constituent entities of the Russian Federation (2020).

The use of the methodology examples of calculating (2020) the relative entropy *H_n RSS_* of regional road safety systems for the best (Kamchatka Territory, Table 4) and the worst (Republic of Tyva, Table 5) from the position of the *H_n RSS_* value of Russian regions is illustrated.

Comparing the best and worst regions of Russia in terms of the orderliness of road safety systems, let us pay attention to the region’s population and the number of road accidents comparable in magnitude, and quantitatively very different regional vehicle fleets, and the number of injured and killed in road traffic accidents. Analysis of the data in Table 4 and Table 5 shows that the fundamental difference between the best and worst constituent entities of the Russian Federation in terms of entropy orderliness of road safety lies in the different transition coefficients *K_i_* of the cause–effect chain “Population *(P)*—The number of vehicles *(N_Vh_*)—The number of road accidents *(N_RA_)*—The number of victims *(N_V_)*—The number of deaths *(N_D_)*” (Table 6).

Note that for the Russian Federation, the values of *K_i_* of the cause–effect chain are intermediate in comparison with the best and worst regions (Table 6).

The best and worst Russian regions in terms of the orderliness of road safety systems differ significantly from each other both in motorization (*K_N_* coefficient) and in the probability of a road accident (*K_RA_* coefficient), in the average number of victims per one accident (*K_V_* coefficient), and by the severity of the outcome of the road accident (*K_D_* coefficient), and this difference is very significant.

Speculations about the reasons for this difference lead us to search for an answer in the social sphere. Table 7 shows some indicators characterizing the social and transport spheres of the two compared regions [33,53].

It is easy to see that the rank analysis characterizes the Republic of Tyva as one of the worst regions of Russia from a social point of view. The Kamchatka Territory is one of the regional leaders. The assessment of the orderliness of the compared regional road safety management systems illustrates this thesis quite clearly.

## 6. Spatio-Temporal Patterns of Changes in the Orderliness of Road Safety Systems in the Russian Federation

### 6.1. Patterns of the Distribution of Regional Values of the Relative Entropy of Road Safety Systems H_n RSS_ in Russia in Space (2020)

Today (2020), the most favorable situation in terms of the orderliness of road safety systems is observed in the economically prosperous regions of the country–in the Ural (*H_n RSS_* = 0.695) and Central (*H_n RSS_* = 0.697) Federal Districts. There is also a fairly high level of orderliness in the field of road safety in the Far Eastern Federal District (*H_n RSS_* = 0.697). On the contrary, the situation is much worse in the economically unfavorable North Caucasian Federal Districts (*H_n RSS_* = 0.736) and Siberian Federal Districts (*H_n RSS_* = 0.716). Other Federal Districts of the country are intermediate in relation to the leaders and outsiders.

The distribution of numerical values of the relative entropy *H_n RSS_* of regional road safety systems in 85 constituent entities of the Russian Federation (2020), shown in Figure 7, is given in Table 8.

Table 9 shows the best three and the worst three regions of the Russian Federation in terms of orderliness of road safety systems.

Let us look at the example from Section 2.2 (Figure 3, Table 2) comparing the quality of regional road safety systems in the neighboring Moscow and Vladimir regions. The characteristic of the level of their orderliness is very different.

The relative entropy of the regional road safety system of the Moscow region *H_n RSS of the Moscow region_* = 0.662; the same for the Vladimir region *H_n RSS of the Vladimir region_* = 0.738. Thus, the Moscow region can be attributed to the regions of Russia with a high level of orderliness of the regional road safety system, and the Vladimir region–to the regions with a low level of road safety orderliness.

Here, as in the case of comparing the Republic of Tyva and the Kamchatka Territory (Table 7), these two regions are at opposite poles in terms of their socio-economic development.

The Moscow region in most positions in Russia takes 2–4th places, while the Vladimir region ranks among the 60–70th [33].

### 6.2. Patterns of Changes in the Relative Entropy of Road Safety Systems H_n RSS_ of the Federal Districts of Russia in Time

Table 10 shows data characterizing changes in the relative entropy of the Russian road safety system *H_n RSS_* in 2004–2020. It is easy to see that, in dynamics (2004–2020), the relative entropy of the road safety system in Russia decreases, which means that its orderliness increases. Over the course of 17 years, the all-Russian value of *H_n RSS_* has decreased by 9.7%.

**Figure 8:**
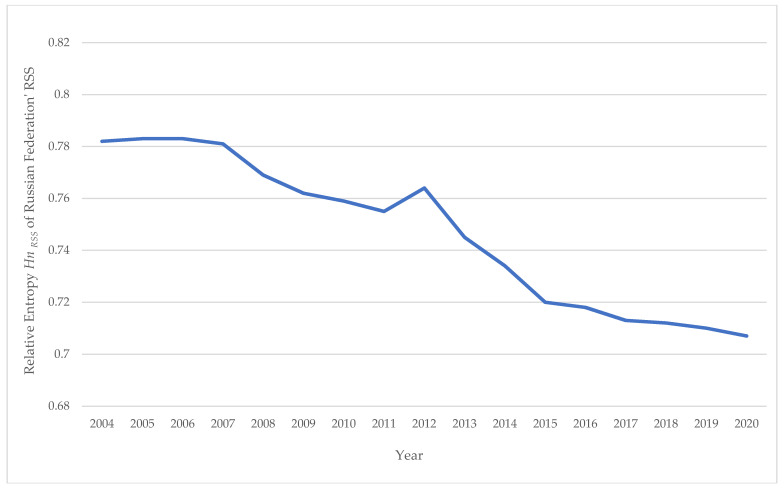
Changes (2004–2020) in the relative entropy of the road safety system *H_n RSS_* of the Russian Federation.

Figure 8 shows the time series (2004–2020) of changes in the relative entropy of the road safety system *H_n RSS_* of the Russian Federation.

In general, the dynamics of the process of decreasing the relative entropy of the Russian road safety system is described by the linear equation *H_n RSS RF_ = 11.9263–0.0056 · Year*. Table 11 shows data on the relative entropy of road safety systems *H_n RSS_* of the Federal Districts of Russia in dynamics in 2004–2020.

**Figure 9:**
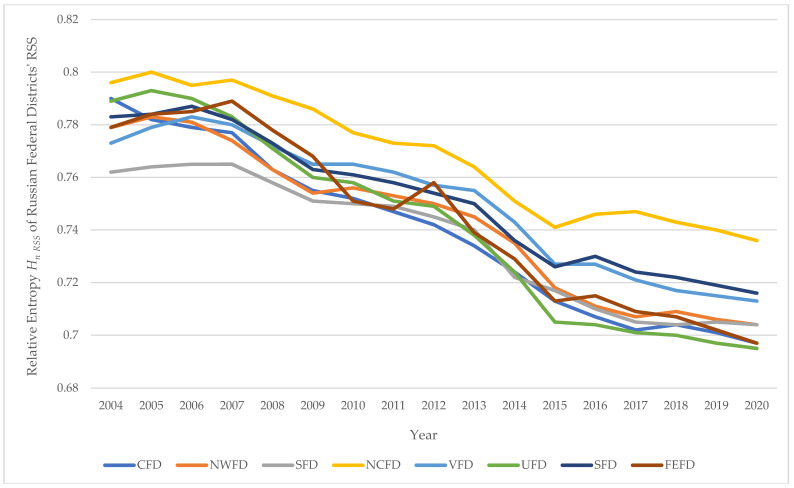
Time series (2004–2020) of the values of the relative entropy *H_n RSS_* of the Federal Districts of the Russian Federation.

Figure 9 shows the corresponding time series of *H_n RSS FD_* values, constructed from the data in Table 11.

Table 12 shows the models of *H_n RSS FD_* = *a–b · Year*, describing the process of changes in time of the relative entropy *H_n RSS_* of the road safety systems of the Federal Districts of the Russian Federation. The data in Table 12 and Figure 9 allow us to conclude that in different regions of Russia the dynamics of improving the orderliness of road safety systems is different. The most important feature that identifies the quality of road safety management is the rate (speed of the process per unit of time) of positive changes in the controlled system.

The conclusion about the quality of road safety management can be drawn from the results of the analysis of the value of the parameter b in the model of *H_n RSS FD_ = a–b · Year*.

Table 13 presents the numerical values of this coefficient for models describing the dynamics of the process in time for the Federal Districts of the Russian Federation.

In the Ural Federal District, the rate of an increase in road safety orderliness is the maximum, and in the North Caucasian Federal District, it is the minimum.

Thus, in terms of the rate of an increase in the orderliness of the road safety systems of the Federal Districts, the best situation is in the Ural Federal District, and the worst is in the North Caucasian Federal District.

This information is very useful precisely for understanding the essence of what is happening in this area and is the basis for taking specific measures at the Federal level of road safety management.

## 7. Discussion of the Results

What determines the dynamics of a decrease in the system entropy *H* and, at the same time, an increase in the orderliness of road safety? First of all, the mentality of humans and their behavior on the road. Discussing the dynamics of orderliness in the field of road safety and stating the positive rate of its growth, we try to find evidence of a positive change in the mentality of the Russian driver. The best tool for this are opinion polls of the population.

**Figure 10:**
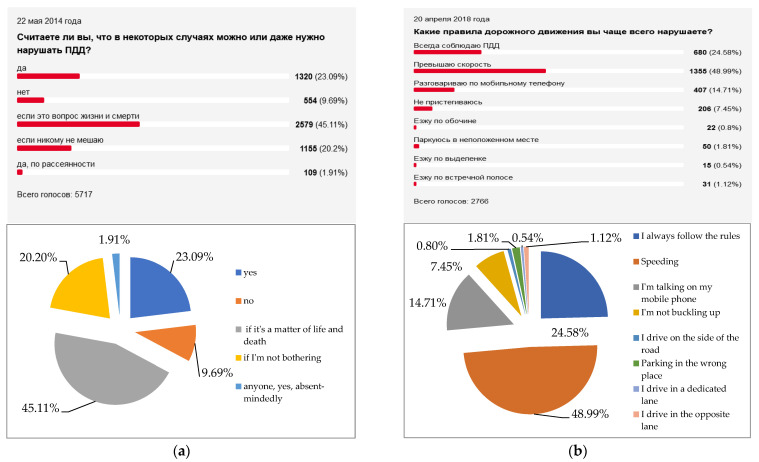
The results of opinion polls regarding the compulsory observance of the road traffic rules by Russian drivers [54]. (**a**) Results as of 22.05.2014 (N = 5717) % of total loyalty to traffic rules = 9.69%. (**b**) Results as of 20.04.2018 (N = 2766) % of total loyalty to traffic rules = 24.58%.

**Figure 11:**
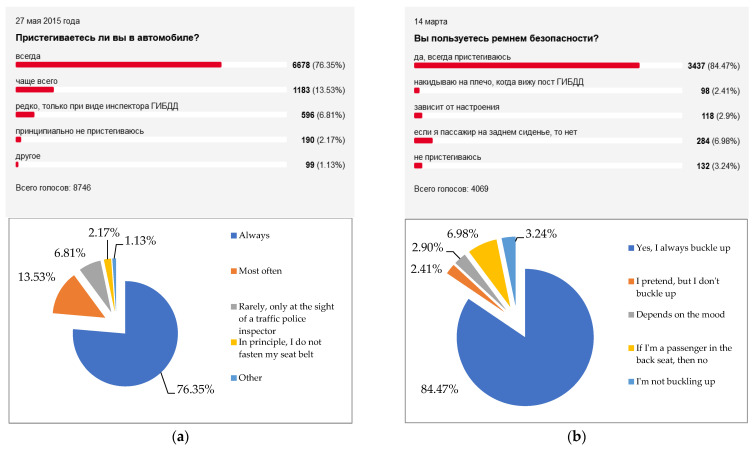
The results of opinion polls regarding the use of seat belts by Russian drivers [54]. (**a**) Results as of 27.05.2015 (N = 8746) % of belt users = 76.35%. (**b**) Results as of 14.03.2019 (N = 4069) % of belt users = 84.47%.

The leading Russian journal for motorists «Za Rulem» [54] quite regularly (from 2012 to the present) conducts opinion polls of its readers. Usually several thousand respondents take part in the polls, and their results are representative. Below are screenshots of the comparative (for different years) results of the distribution of answers to two most important questions—about the attitude to the compulsory observance of the Road Traffic Rules (Figure 10) and the use of seat belts (Figure 11).

It is easy to see that over time, the proportion of Russian drivers who obey the law and practice safe behavior is gradually increasing. This largely explains the general tendency towards a decrease in the level of entropy of road safety systems *H_n RSS_*. The regional diversity of this general trend is explained by the effect of many other factors, such as the quality of the vehicle fleet [55], the quality of the road transport infrastructure [56], and the general quality of life of the population [57].

## 8. Conclusions

In conclusion, I would like to point out the main results obtained in the course of the research.

First, the entropy method can be successfully used in road safety management in large federal countries with a complex administrative structure. The continuous complication of transport systems, the growth of automobilization, and people’s transport mobility require more professional system management. This issue has not been successfully solved in every case. As a consequence, high heterogeneity of the quality of the regional transport system functioning, including the road safety provision sphere, occurs. The problem has especially manifested itself in large countries (Russian Federation, USA, China, Brazil, Canada, Australia, etc). Nowadays, the problem of the absence of the methodology of the fair, from the regions’ view, road safety management clearly manifests itself at the federal level in Russia. Primarily, it concerns the distribution of the resources, required for the provision of high-level road safety, between regions. Another problem, typical for road safety management in large federal countries, is the necessity of the proper and professional statement of different target levels for different countries’ regions. The choice of suitable methodological approaches of road safety provision for certain places is also important. That is the exact purpose of federal road safety management. However, for now, federal authorities do not have ideal instruments for the comparison of road safety spheres in different country’s regions.

Second, it is the indicator of relative entropy *H_n RSS_* that makes it possible to quantify the orderliness of the regional road safety system. This indicator has an important advantage–it can be used to identify the structural perfection of the road safety system, which allows us to compare systems that are completely different in size. Another significant advantage of this indicator over other characteristics of the assessment of road safety, such as social risk, transport risk, and the coefficient of road accident severity, is complexity. This indicator embodies all advantages of other characteristics of road safety assessment.

Third, the assessment of the relative entropy *H_n RSS_* of the Russian regional road safety systems shows that the orderliness in the road safety sphere in Russia varies over a wide range of values of *H_n RF RSS 2020_* = [0.645; 0.826], and the rate of its decline in 2004–2020 varies for different regions of the country. It is an important conclusion that allows one to both comprehensively and differentially for regions assess the general situation in the sphere of the road safety provision in Russia. The knowledge of general and partial regularities of changes of relative entropy *H_n RSS_* of Russian regional road safety provision systems in time and space allows one to develop and use differentiated approaches for different regions in federal management.

Fourth, the leaders and outsiders among Russian regions were identified in terms of the current level of orderliness of regional road safety systems and in terms of the rate of its increase. In particular, in recent years, the Ural Federal District is the leader of the rate of increase in the orderliness of regional road safety provision systems. Vice versa, the North Caucasian Federal District is the outsider in this sphere. The analytics of the current situation (2020) of the road safety orderliness in administrative regions showed that Kamchatka Krai is leading (*H_n Kamchatka RSS 2020_* = 0.645) and the Tyva Republic has the worst result (*H_n Tyva RSS 2020_* = 0.826).

Fifth, a methodology for road safety management in large-scale systems using the entropy method was developed. The algorithm of the management of the regional road safety in Russia concerning the level of its entropic orderliness is presented in Section 4.

Sixth, an attempt was made to explain the trends of increasing orderliness in the field of road safety by positive changes in the mentality of Russian drivers. I believe that this is an essential conclusion of the research because it is always worth knowing what generates positive effects in the state of the managed systems. Statistics presented in the article partially demonstrate that, in recent years, the transport culture of road traffic participants has gradually improved in Russia.

Most likely, the next step in my study will be measuring the predictability and complexity of time series using entropy [58].

Summarizing what is above, I would like to express hope for a gradual improvement of the road safety situation in Russia. Despite the fact that today Russia significantly lags behind the world leaders in road safety–the Scandinavian countries, Great Britain, Ireland, and Switzerland (Table 1)–it is still moving along a positive path to reduce chaos in transport systems and improve orderliness in the field of road safety. This is evidenced by the results of the studies. The use of the entropy method of road safety management can accelerate this process.

## Figures and Tables

**Table 1:** Duration of the period of reduction of Human Risk (*HR*) from level 11 to level 4 road deaths/100 thousand people [12,13].

Country	Duration of the Period of *HR* Reduction from 11 to 4 RA Fatalities /100,000 People	Year of Reaching the *HR* Level of 11 RA Fatalities /100,000 People	Year of Reaching the *HR* Level of 4 RA Fatalities /100,000 People	Current (2018) *HR* Level, RA Fatalities /100,000 People
Norway	30	1978	2008	2.0
Switzerland	20	1991	2011	2.7
Great Britain	32	1977	2009	2.8
Ireland	12	2000	2012	2.9
Denmark	19	1992	2011	3.0
Sweden	29	1980	2009	3.2
Japan	36	1978	2014	3.3
Estonia	12	2005	2017	3.6
Netherlands	25	1984	2009	3.9
Spain	10	2003	2013	3.9
Germany	27	1989	2016	4.0

**Table 2:** Actual socio-economic living conditions of the population and the level of road accident rate in the neighboring regions of Russia in 2019 [9,32,33].

Geographically Adjacent Regions of the Russian Federation	Characteristics of the Socio-Economic Development of Regions [33]				Road Accident Rate Characteristics [9,32]		
	Gross Regional Product, Thous. Rubles per Person /Year	Average per Capita Income of Population, Thous. Rubles per Person /Month	Motorization, Vehicle/1000 People	Density of Highways, km/km^2^	Human Risk, Road Deaths /100 Thous. People	Transport Risk, Road Deaths /100 Thous. Vehicles.	RTA Severity, RTA Fatalities /100 Injured in RTA
Vladimir region	394.6	25.3	366	0.522	19.08	52.12	7.69
Moscow region	670.8	47.2	416	0.972	12.50	30.01	11.44

**Table 3:** Intermediate stages and the final result of calculating the relative entropy *H_n RSS_* of the road safety system of the Russian Federation (2020).

Population (*P*) in the Russian Federation (2020), People [9]	Fleet of Vehicles (*Vh*) (2020), Vehicles [9]	Number of Road Accidents (*RA* *Number*) (2020), Units [9]	Number of Victims (*V* *Number*) in Road Accidents (2020), People [9]	Number of Deaths (*D* *Number*) in Road Accidents (2020), People [9]
146,888,583	58,992,696	137,655	190,965	15,788
**Values of coefficients of information transmission between links of the cause–effect chain**				
*K_N_*		*K_RA_*	*K_V_*	*K_D_*
0.4016		0.0023	1.3873	0.0827
**Positive of a link in the cause-and-effect chain**$Q_{i}=\ln\left(1/K_{i}\right)$ if *K_i_* < 1 or $Q_{i}=\ln\left(K_{i}\right)$ if *K_i_* > 1				
0.9123		6.0604	0.3273	2.4928
**Relative weight of positive of a corresponding link in the chain** $w_{i}$				
0.0932		0.6189	0.0334	0.2546
**Estimated value [** $\ln\;(w_{i})$ **]**				
−2.3735		−0.4799	−3.3984	−1.3682
**Estimated value [** $w_{i}$ $\cdot \ln\;(w_{i})$ **]**				
−0.2211		−0.2970	−0.1136	−0.3483
**Estimated entropy value**$H=-\sum_{i=1}^{n}w_{i}\cdot \ln w_{i}$ = 0.980				
**Estimated value of relative entropy**$H_{n}{\;}_{RSS\;}=H/H_{\max}=H/\ln(4)$ = 0.707				

**Table 4:** Intermediate stages and the final result of calculating the relative entropy *H_n RSS_* of the road safety system of the Kamchatka Territory (2020)–the best region of the Russian Federation for this indicator.

Population (*P*) in the Kamchatka Territory (2020), People [9]	Fleet of Vehicles (*Vh*) (2020), Vehicles [9]	Number of Road Accidents (*RA* *Number*) (2020), Units [9]	Number of Victims (*V* *Number*) in Road Accidents (2020), People [9]	Number of Deaths (*D* *Number*) in Road Accidents (2020), People [9]
311,667	213,559	471	678	47
**Values of coefficients of information transmission between links of the cause–effect chain**				
*K_N_*		*K_RA_*	*K_V_*	*K_D_*
0.6852		0.0022	1.4395	0.0693
**Positive of a link in the cause-and-effect chain**$Q_{i}=\ln\left(1/K_{i}\right)$ if *K_i_* < 1 or $Q_{i}=\ln\left(K_{i}\right)$ if *K_i_* > 1				
0.3780		6.1168	0.3643	2.6690
**Relative weight of positive of a corresponding link in the chain** $w_{i}$				
0.0397		0.6420	0.0382	0.2801
$\mathrm{Estimated}\;\mathrm{value}\;[\ln\;(w_{i})$]				
−3.2271		−0.4432	−3.2641	−1.2725
$\mathrm{Estimated}\;\mathrm{value}\;[w_{i}$$\cdot \ln\;(w_{i})$]				
−0.1280		−0.2845	−0.1248	−0.3565
$\mathrm{Estimated}\;\mathrm{entropy}\;\mathrm{value}\;H=-\sum_{i=1}^{n}w_{i}\cdot \ln w_{i}$ = 0.894				
$\mathrm{Estimated}\;\mathrm{value}\;\mathrm{of}\;\mathrm{relative}\;\mathrm{entropy}\;H_{n}{\;}_{RSS\;}=H/H_{\max}=H/\ln(4)$ = 0.645				

**Table 5:** Intermediate stages and the final result of calculating the relative entropy *H_n RSS_* of the road safety system of the Republic of Tyva (2020)–the worst region of the Russian Federation for this indicator.

Population (*P*) in the Republic of Tyva (2020), People [9]	Fleet of Vehicles (*Vh*) (2020), Vehicles [9]	Number of Road Accidents (*RA* *Number*) (2020), Units [9]	Number of Victims (*V* *Number*) in Road Accidents (2020), People [9]	Number of Deaths (*D* *Number*) in Road Accidents (2020), People [9]
330,368	70,452	472	855	101
**Values of coefficients of information transmission between links of the cause–effect chain**				
*K_N_*		*K_RA_*	*K_V_*	*K_D_*
0.2133		0.0067	1.8114	0.1181
**Positive of a link in the cause-and-effect chain**$Q_{i}=\ln\left(1/K_{i}\right)$ if *K_i_* < 1 or $Q_{i}=\ln\left(K_{i}\right)$ if *K_i_* > 1				
1.5453		5.0057	0.5941	2.1360
**Relative weight of positive of a corresponding link in the chain** $w_{i}$				
0.1665		0.5393	0.0640	0.2301
$\mathrm{Estimated}\;\mathrm{value}\;[\ln\;(w_{i})$]				
−1.7928		−0.6174	−2.7486	−1.4691
$\mathrm{Estimated}\;\mathrm{value}\;[w_{i}$$\cdot \ln\;(w_{i})$]				
−0.2985		−0.3330	−0.1760	−0.3381
$\mathrm{Estimated}\;\mathrm{entropy}\;\mathrm{value}\;H=-\sum_{i=1}^{n}w_{i}\cdot \ln w_{i}$ = 1.146				
$\mathrm{Estimated}\;\mathrm{value}\;\mathrm{of}\;\mathrm{relative}\;\mathrm{entropy}\;H_{n}=H/H_{\max}=H/\ln(4)$ = 0.826				

**Table 6:** The fundamental difference between the numerical values of the information transmission coefficients between the links of the cause–effect chain of the road accident rate formation (2020).

Compared Road Safety Systems	*H_n RSS_*	Values of Information Transfer Coefficients between Links of the Cause–Effect Chain			
		*K_N_*	*K_RA_*	*K_V_*	*K_D_*
Kamchatka Territory	0.645	0.6852	0.0022	1.4395	0.0693
Republic of Tyva	0.826	0.2133	0.0067	1.8114	0.1181
Russian Federation	0.707	0.4016	0.0023	1.3873	0.0827

**Table 7:** Comparison of the best and worst constituent entities of the Russian Federation in terms of orderliness of regional road safety systems (2020) [33,53].

Indicator	Indicator Values/Rank № Out of 85		
	Kamchatka Territory	Russian Federation	Republic of Tyva
**Characteristics of the socio-economic sphere [33]**			
Gross regional product, thous. rubles/person per year	891.0/11	735.1/-	243.1/80
Specific volume of expenditures of the budget of a constituent entity of the Russian Federation, thous. rubles/person per year	309.3/9	156.9/-	128.2/63
Unemployment, % of the labor force	3.8/5	5.8/-	18.0/82
**Characteristics of regional transport systems [53]**			
Motorization of the population, vehicles/1000 people	685.2/1	401.6/-	213.3/85
Estimated traffic density, vehicle/1 km	94.5/5	33.2/-	7.9/84
Transport Risk *TR*, road deaths/100 thous. vehicles	22.0/17	26.8/-	143.4/85

**Table 8:** Distribution of regions of the Russian Federation by value ranges of the relative entropy *H_n RSS_* of regional road safety systems (2020).

The Number of Regions of the Russian Federation (85 in Total) for Whose Road Safety Systems the Relative Entropy *H_n RSS_* of Regional Road Safety Systems Is in the Value Ranges									
[0.645; 0.660]	[0.661; 0.680]	[0.681; 0.700]	[0.701; 0.720]	[0.721; 0.740]	[0.741; 0.760]	[0.761; 0.780]	[0.781; 0.800]	[0.801; 0.820]	[0.821; 0.826]
7	10	13	21	25	6	2	-	-	1
**Actual (2020) orderliness of regional road safety systems**									
High		Average		Low		Very low			

**Table 9:** The best and worst regions in Russia by the value of the relative entropy *H_n RSS_* (2020).

Regions of the Russian Federation			
The Best in Terms of Orderliness of Road Safety Systems	*H_n RSS_*	The Worst in Terms of Orderliness of Road Safety Systems	*H_n RSS_*
Kamchatka Territory	0.645	Republic of Tuva	0.826
Pskov Region	0.655	Republic of Ingushetia	0.778
Sverdlovsk Region	0.657	Karachayevo-Circassian Republic	0.773

**Table 10:** Changes in the relative entropy *H_n RSS_* of the Russian road safety system in 2004–2020.

System	Value Relative Entropy of Road Safety Systems *H_n RSS_* in the Russian Federation/Years							
	2004	2005	2006	2007	2008	2009	2010	2011
Russia	0.782	0.783	0.783	0.781	0.769	0.762	0.759	0.755
**Value relative entropy of road safety systems *H_n RSS_* in the Russian Federation/Years**								
**2012**	**2013**	**2014**	**2015**	**2016**	**2017**	**2018**	**2019**	**2020**
0.764	0.745	0.734	0.720	0.718	0.713	0.712	0.710	0.707

**Table 11:** Changes in the relative entropy of road safety systems *H_n RSS_* of the Federal Districts of Russia in dynamics in 2004–2020.

Year	Value Relative Entropy of Road Safety Systems *H_n RSS_* in the Federal Districts of Russian Federation							
	*CFD*	*NWFD*	*SFD*	*NCFD*	*VFD*	*UFD*	*SFD*	*FEFD*
2004	0.790	0.779	0.762	0.796	0.773	0.789	0.783	0.779
2005	0.782	0.783	0.764	0.800	0.779	0.793	0.784	0.784
2006	0.779	0.781	0.765	0.795	0.783	0.790	0.787	0.785
2007	0.777	0.774	0.765	0.797	0.780	0.783	0.782	0.789
2008	0.763	0.763	0.758	0.791	0.772	0.771	0.773	0.778
2009	0.755	0.754	0.751	0.786	0.765	0.760	0.763	0.768
2010	0.752	0.756	0.750	0.777	0.765	0.758	0.761	0.751
2011	0.747	0.753	0.749	0.773	0.762	0.751	0.758	0.748
2012	0.742	0.750	0.745	0.772	0.757	0.749	0.754	0.758
2013	0.734	0.745	0.740	0.764	0.755	0.738	0.750	0.739
2014	0.724	0.735	0.722	0.751	0.743	0.724	0.736	0.729
2015	0.713	0.718	0.717	0.741	0.727	0.705	0.726	0.713
2016	0.707	0.711	0.710	0.746	0.727	0.704	0.730	0.715
2017	0.702	0.707	0.705	0.747	0.721	0.701	0.724	0.709
2018	0.704	0.709	0.704	0.743	0.717	0.700	0.722	0.707
2019	0.701	0.706	0.705	0.740	0.715	0.697	0.719	0.702
2020	0.697	0.704	0.704	0.736	0.713	0.695	0.716	0.697

Note. CFD—Central Federal District; NWFD—North-Western Federal District; SFD—Southern Federal District; NCFD—North Caucasus Federal District; VFD—Volga Federal District; UFD—Ural Federal District; SFD—Siberian Federal District; FEFD—Far Eastern Federal District.

**Table 12:** Models of *H_n RSS FD_ = a–b · Year* for the road safety systems of the Federal Districts of the Russian Federation.

Federal Districts of Russian Federation	Model of *H_n RSS FD_ = a–b · Year*
North Caucasus Federal District (NCFD)	*H_n RSS NCFD_ = 9.8318–0.0045 · Year*
Southern Federal District (SFD)	*H_n RSS SFD_ = 10.1157–0.0047 · Year*
Volga Federal District (VFD)	*H_n RSS VFD_ = 10.4355–0.0048 · Year*
Siberian Federal District (SFD)	*H_n RSS SFD_ = 10.6730–0.0049 · Year*
North-Western Federal District (NWFD)	*H_n RSS NWFD_ = 11.9469–0.0056 · Year*
Central Federal District (CFD)	*H_n RSS CFD_ = 13.3390–0.0063 · Year*
Far Eastern Federal District (FEFD)	*H_n RSS FEFD_ = 13.4079–0.0063 · Year*
Ural Federal District (UFD)	*H_n RSS UFD_ = 14.9835–0.0071 · Year*

Note. The order of presentation of the Federal Districts in Table 12 is determined by the rank of the rate of change of the relative entropy *H_n RSS FD_* (according to the value of the parameter b of the model)–from the lowest to the highest.

**Table 13:** Identification of the rate and quality of the process of dynamics of the orderliness of road safety systems of the Federal Districts of Russia in 2004–2020.

Value of the Parameter *b* in the Model of *H_n RSS FD_ = a–b · Year* for the Federal Districts of the Russian Federation							
*CFD*	*NWFD*	*SFD*	*NCFD*	*VFD*	*UFD*	*SFD*	*FEFD*
0.0063	0.0056	0.0047	0.0045	0.0048	0.0071	0.0049	0.0063
**Quality of the process of dynamics of the orderliness of road safety systems**							
Above average	Average	Below average	Low	Below average	High	Below average	Above average

Note. The quality of the dynamics process is identified by the value of the parameter b: ≤ 0.0045–low; 0.0046 … 0.0055–below average; 0.0056 … 0.0060–average; 0.0061 … 0.0070–above average; ≥ 0.0071–high.

## Data Availability

Not applicable.
